# Strategies and Measurement Tools in Physical Activity Promotion Interventions in the University Setting: A Systematic Review

**DOI:** 10.3390/ijerph17186526

**Published:** 2020-09-08

**Authors:** David García-Álvarez, Raquel Faubel

**Affiliations:** 1 Faculty of Physiotherapy, University of Valencia, 46010 Valencia, Spain; dagaral4@alumni.uv.es; 2 Department of Physiotherapy, University of Valencia, 46010 Valencia, Spain; 3 Joint Research Unit in Biomedical Engineering (eRPSS: IIS La Fe-UPV), 46026 Valencia, Spain

**Keywords:** physical activity, students, healthy universities, university, setting approach, health promotion

## Abstract

The university environment is especially suitable for implementing health promotion interventions and specifically for physical activity promotion among university students. The objective of this systematic review was to describe the strategies employed and the physical activity data collection tools that have been used in said interventions. A systematic search for articles was conducted using the PubMED, Cochrane, and PEDro databases. The articles selected were those describing a physical activity promotion intervention aimed at university students in their own university setting in which there was a control group. Eventually, 1074 articles were identified, of which 13 fulfilled the selection criteria. The results show eight strategies and nine different instruments for collecting physical activity data. The strategies identified were used in combination and they were adapted in each of the complex interventions. Validated questionnaires were the most widely used instrument. Future original studies are needed to find out the impact of these strategies in physical activity promotion among university students specifically in the university context.

## 1. Introduction

In 2010, the data and figures extracted by the World Health Organization (WHO) [1] indicated that approximately 23.3% of the world adult population—updated to 27.5% in 2016 [2]—and 81% of adolescents did not reach the minimum levels of physical activity necessary to gain some health benefit. Some authors have even maintained in their studies that physical inactivity is responsible for a quarter of global mortality [3], which raises the urgent need to promote lifestyles including physical activity among the global population. The same data and figures from the WHO indicate that only 56% of member states of the organization have implemented physical activity promotion policies.

There is a striking difference between the inactive percentages of the adult and adolescent populations, which is even more alarming considering that adolescence (from 9 to 18 years of age) is the period when health behaviours develop and said behaviours significantly predict the physical activity that these subjects will carry out as adults [4]. 

As the adolescent population is so vulnerable, it is not surprising that it should be the subject of most physical activity promotion interventions. However, in addition to the above, the fact that it has been observed that around the age of 15 there is a reduction in physical activity, among both the male and female population [5], combined with the fact that during the first year of university there is another significant decrease [6,7], means that there is another highly vulnerable group, not just due to its lower level of physical activity, but also as a continuation of the interventions carried out during adolescence, which have not been able to show maintained results in the long term [8].

This population shows characteristics that make them easier to work with and interventions that have been carried out show favourable results. It also has some disadvantages, such as the complicated organisational structure of universities, which makes it difficult to implement large-scale interventions, and higher education aims do not systematically prioritise health and well-being [9], which is in fact the case in pre-university educational institutions. One of the advantages offered by these centres is that it has been observed that the university environment is one in which students do not just receive an education, but also develop personally and socially. This development has a great influence on students during their time at university and also during the rest of their lives, affecting the choices they will make, their values and priorities, their jobs, homes, and communities [10]. Furthermore, from a health perspective, the educational environment of universities offers a great variety of assets that can be used to promote physical activity, such as their own facilities, teaching staff, the university’s own programmes, or economic collaboration.

This gave rise to the strategy of Health Promoting Universities, universities committed to promoting health in the university environment, which “aspire to create a learning environment and organisational culture that improves health, well-being, and sustainability in its community, and allows people to reach their maximum potential” [11]. In Spain, The Spanish Network of Healthy Universities (REUS), founded in 2008, includes universities across the country, as well as: The Conference of Rectors of Spanish Universities; the Ministry of Health, Social Services and Equality; the Ministry of Education, Culture and Sport; and some autonomous public health structures [12]. 

Some researchers have studied the effectiveness of health promotion interventions on university students, mainly on food, weight control, and stress, as well as physical activity [13,14]. However, there is not enough scientific evidence on how said strategies are implemented in the university setting, which strategies are involved, and the instruments for measuring physical activity in this context. 

The general objective of this systematic review is to compile and evaluate current existing evidence on physical activity promotion interventions in the university setting and to describe the different strategies and the tools used to collect variables related to physical activity.

## 2. Materials and Methods

This study follows the guidelines of the Preferred Reporting Items for Systematic reviews and Meta-Analysis (PRISMA) [15]. 

### 2.1. Selection of the Studies

The articles included were those published in any language from 2013 to 2018 (both inclusive) that carried out a physical activity promotion intervention aimed at university students in their own university environment in which there was a control group. Studies were excluded if they were carried out on subjects with a specific disease or with participants under the age of 18, as well studies with monitoring time less than 3 months. All of the identified articles were independently analysed by two researchers from the present study and the final selection of the articles to be included was made by consensus.

### 2.2. Research Strategies

The systematic search was carried out in PubMED, PEDro, and Cochrane. The search in PubMED and Cochrane was: ((physical activity OR exercise) AND (health promotion OR health promoting effects) AND (university student OR college student OR young adults)) and ((physical activity promotion OR exercise promotion) AND (university student OR college student OR young adults)). The following keywords were used in PEDro: health promotion, physical activity, college students, university students, young adults. A manual search was also carried out, including the references of the articles found and related articles.

### 2.3. Data Extraction 

The variables included in Tables 1 and 2 were gathered, such as country of implementation and year of publication, population, measurement time, strategies carried out in the intervention group and control group, and tools for collecting information on physical activity.

**Figure 1:**
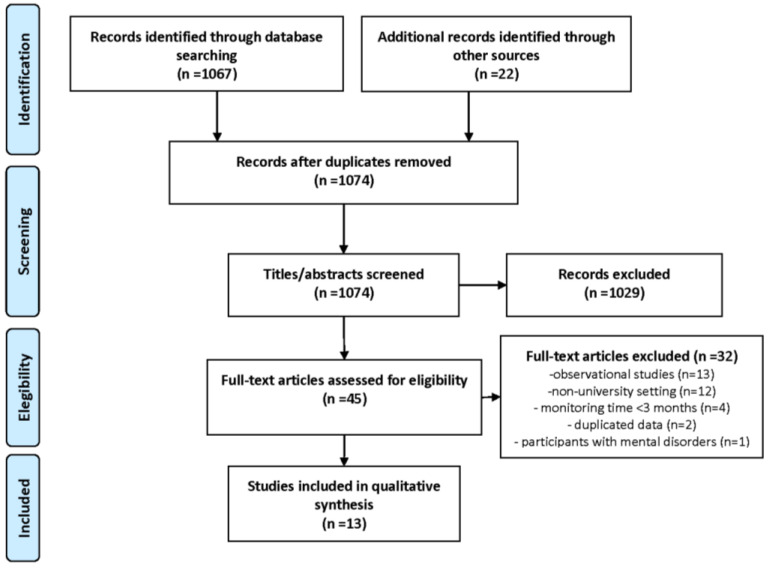
Flowchart of the study selection process.

As shown in the PRISMA flow diagram (Figure 1), after the initial search and eliminating duplicates, 1074 articles were identified, of which 1029 were eliminated after reading the title and summary. Of the 45 remaining, after critical reading of the complete text, 32 other articles were rejected and 13 studies were finally selected for inclusion in the systematic review. Among the 13 articles selected, 10 were randomized control trials (RCT) with methodological quality assessed using the Jadad scale. The methodological quality of the other 3 articles was assessed through critical appraisal.

## 3. Results

### 3.1. Descriptive Characteristics of the Included Studies

The 13 studies included were carried out in seven different countries: Canada [16,17], United States [18,19,20,21,22,23], Japan [24], South Korea [25], United Kingdom [26], South Africa [27], and Thailand [28], with a total of 7193 participants. The majority of the studies measured physical activity per week, although some included other variables of interest regarding physical activity like steps per day [23,28], physical activity per month [23], leisure time physical activity [17,18,28], going to the gym [20,21], outcomes expectancies [16,28], intention of carrying out physical activity [24], physical activity action planning [16], motivation to be active [20,23], state of change [23,24] and physical activity enjoyment [23]. 

The monitoring periods for each study varied from 3 to 15 months. The smallest sample included in this review has 77 subjects [24], compared to the 1639 participants included in the study by Kattelmann et al. [19] and the 2614 participants in the study carried out by Cameron et al. [26]. Table 1 summarizes the most relevant data from the articles included.

### 3.2. Strategies for Physical Activity Promotion in the University Setting

Each study included in the review applies different strategies for physical activity promotion in the university setting. These strategies have been classified into eight broad categories of strategies and are described in Table 2: (a) health promotion courses (b) periodic messages, (c) online profile creation, (d) physical activity, exercise, or sports programs attendance, (e) pedometer or activity tracker, (f) incentives to attend fitness center, (g) training of physical activity courses teachers, and (h) pre-test sensitization.

### 3.3. Data Collection Instruments

Throughout the 13 articles included in this review, nine different methods were used for evaluating the physical activity carried out by the subjects during each of the measurement periods, including questionnaires, self-recording of activity, and direct methods (accelerometers or pedometers) and identification cards to record attendance at sports centres.

Five questionnaires for assessment of physical activity were used, including the Godin-Shephard Leisure-Time Physical Activity Questionnaire in three studies [17,18,28]; the International Physical Activity Questionnaire in its short version in another three studies [23,25,26] and the long version in two studies [19,24]; and one study [16] used the Global Physical Activity Questionnaire. One of the studies used a questionnaire including three open-ended questions to record physical activity [27].

The self-recording methods include the 30-Day Physical Activity Recall (30-Day PAR) and the 8-response physical activity self-reporting measure (PA-8), both used in the same article [23]. Other instruments used for direct collection of variables related to carrying out physical activity were use of pedometers and accelerometers [17,22,23,28] and recording of attendance at sports centres with identification cards [20,21]. 

## 4. Discussion

This systematic review identified 1074 articles, of which 13 fulfilled the selection criteria. The results show diverse strategies implemented in university environments, grouped in 8 categories and carried out in combination to promote physical activity among university students. Thus, nine different instruments for collecting physical activity data have been identified.

In the studies included in this review, complex interventions were implemented, combining different strategies, as shown in Table S1, except for Pope et al. [20,21], who solely implemented incentives to attend sports centers. Thus, the strategies have been described separately for each study, although the effectiveness of the intervention is due to the synergy between the different strategies implemented and not the isolated effect of a single strategy. Educational training on health promotion and physical activity promotion is the more used strategy in the selected studies. It was developed face-to-face [16,17,18,24,25,27] or online [19,26,28] in order to raise awareness of their importance in health promotion and disease prevention. Furthermore, during these courses, participants were encouraged to set themselves short- and medium-term goals and to fulfil them as a method of adherence to healthy lifestyles. Sending messages regarding health promotion/disease prevention and physical activity generally includes audiovisual content to encourage people to engage in physical activity. This strategy varied in each study, including (a) sending a weekly message to increase walking time [25], (b) following the theory of planned behaviour with messages that were shown to the participants as they fulfilled goals in the web profile of the intervention [26], (c) reinforcing online training with three personalised messages per week and 1 programme loyalty message [19], (d) sending three monthly messages to reinforce the use of the pedometer, with tips to increase the number of daily steps, encouraging them to fulfil minimum daily objectives and informing them of the benefits of doing physical activity on campus [17], and (e) a weekly message encouraging them to continue with the programme [28]. This strategy is combined in some studies with strategies based on the use of an online profile and self-management of participation on a website or application [19,22,23,24,26,28]. Participants could use this profile to follow the goals suggested by the authors, to set up their own goals, and check their progress, using their results as feedback to continue. They could include their motivations for continuing with physical activity in these profiles as a reinforcement. 

An explicit setting approach in health promotion is implemented in the intervention developed by Bang et al. [25], organising group walks in the university environment during the lunch hour. In this line, other studies [16,18,27] proposed physical exercise sessions or sporting competitions, sometimes combined with theoretical training courses on healthy lifestyles.

Pedometers and activity trackers have been used as a strategy for physical activity promotion itself, not just as a data collection tool. In one of the studies [17], the subjects merely had to carry the pedometer with them every day, although in the majority of the studies [22,23,28], it was used in combination with online applications in such a way that the participants could see in real time the number of steps they had done and they could set themselves new objectives on that basis. A different concept was used in another strategy based on weekly incentives: this intervention consists of encouraging the participants to go to the gym or sports centres by giving them an amount of money every week (in these cases, a low amount) if they fulfilled the objectives. Only two studies included this type of intervention and both were carried out by Pope et al. in 2013 [21] and 2015 [20]. In the first study, weekly incentives were given if they fulfilled the objectives, starting with 5 dollars and increasing by $0.25 up to a maximum of $7.75. In the 2015 study, they added another group that, in addition to receiving the same incentives during the first semester of the course, received incentives during three random weeks during the second semester if they had been fulfilling the objectives.

Regarding the data collection instruments, The Godin-Shephard Leisure-Time Physical Activity Questionnaire [29] attempts to classify the number of times that people do sessions of at least 15 min (although some variations use a period of 30 min) of mild, moderate, or intense physical activity every week in their leisure time, giving a value of 3, 5, or 9 metabolic equivalents (METs), respectively, to each period of each intensity and calculating a total number of METs at the end of the week. The short form of the International Physical Activity Questionnaire [30] calculates the total METs used in physical activity at the end of the week. In this case, it is a questionnaire of nine items that divides time into generally active time and sedentary time and it is the subject who reports the minutes dedicated to each activity. It classifies activity as vigorous, moderate, or walking, allocating 8 METs/min (intense activity), 4 METs/min (moderate activity), and for walking, 5, 3.3, or 2.5 METs/min, depending on the intensity. Sedentary time is always allocated 1 MET/min. The long form of the International Physical Activity questionnaire [31] includes 31 items and classifies physical activity not just by its intensity, but also by the context in which it is carried out (work, transport, domestic and gardening activities, and leisure time). The Global Physical Activity Questionnaire [32] endorsed by WHO comprises three sections that assess the physical activity carried out during a typical week day evaluating (a) the moderate to vigorous physical activity carried out at work, (b) during transport, and (c) activity carried out during leisure time. In total, the questionnaire consists of 16 questions that ask about the frequency and duration of each activity, including sedentary behaviour. Finally, another study uses a questionnaire made up of three open questions in a survey produced by the Center for Disease Control and Prevention in the United States to verify the quantity and intensity of physical activity carried out by participants over the last 7 days. 

One of the self-recording measures, the 30-Day PAR, asks the subjects to recall and record physical activity carried out over the last 30 days [33]. Whereas in the PA-8, the subjects select from one of the 8 possible responses that best fits their level of physical activity. The responses are graduated in such a way that selecting the fifth response or higher indicates a sufficient level of physical activity to fulfil the recommendations [34]. 

Of all the data collection instruments, the most commonly used in the articles included in this review was validated questionnaires on physical activity, used by 9 of the 13 studies, partly thanks to its proven reliability and the reproducibility of results [29,30,31]. One of the benefits of using questionnaires, in comparison with other methods such as using pedometers or accelerometers, is the low level of influence that it has on the results. This review shows that a pedometer or accelerometer could be used as an intervention in itself to promote physical activity, while using questionnaires does not have a significant effect, as demonstrated by the study that used pre-test sensitization [28] without finding positive results. Limiting the use of pedometers or accelerometers to just specific weeks (one week at the beginning, one at the end, and at times, in the middle of the follow-up period) is a tool for reducing its impact on results. However, this also reduces the capacity to identify variations in physical activity throughout the year due to the influence of external factors such as exam periods [35,36,37] or season [38,39]. Use of questionnaires could also offer advantages in comparison with gym identification cards. Normally, those were used in conjunction with monitoring inside the gym to prevent participants from using them to get in but not carrying out any activities once inside. Nevertheless, gym cards do not provide data on the quality and intensity of exercise carried out by the subjects. 

A recent study [14] systematically reviewed the effectiveness of physical activity promotion interventions carried out among university students in any context (a university or non-university context). This review describes the strategies and behaviour change techniques used in the interventions. However, the results of the review show that the level of evidence regarding the immediate and the long-term effects of interventions to promote physical activity among university students is limited. Another previous review published in 2015 [13] also analysed the effectiveness results of different interventions not just targeting physical activity, but also nutrition and weight-loss behaviours amongst university and college students. This review shows that 18/29 studies examining physical activity found significant effects on physical activity. In line with our review, this study also highlights the importance of the setting approach since universities and colleges are an ideal setting for implementation of health promotion programmes. This is related to the health assets that can be found in the university environment, both in terms of facilities and opportunities and in terms of university community staff (particularly linked to health disciplines). Likewise, it is during university that lifestyle skills and behaviours are developed and established. 

In line with previous studies [13,14], the effectiveness of interventions implemented for physical activity promotion in a university setting included in this review (Table S2) is variable, with 70% of the studies reporting significant improvement in a variable related to physical activity as MVPA and physical activity action planning [16], gym attendance [20,21], steps/day [23,28], SOC variables [28], state of change [24], or leisure time physical activity [28]. In some studies, significant differences have been found just in specific types of physical activity [19] or for specific populations (i.e., women [19] or subjects non-engaged in sports [24]). Annesi et al. [18] and Kattelmann et al. [19] have found significant differences between baseline and follow-up (within group analysis) in the intervention group, but also in the control group. In our review, four studies [17,22,25,26] found that the intervention group did not experience any significant increase in their level of physical activity or even that this group actually reduced their level of physical activity during the university semester [21,22,23]. 

Previous studies have analysed the possible influence of exam periods on levels of physical activity due to the increase in inactivity and sedentary behaviours [35,36,37,39,40]. These studies show that between 41.42% and 44.58% of a university student’s total sedentary time (an average of 4.6 hours/day during the week during exam periods) is spent studying [40,41] and with the increase in tasks related to exam periods, students prefer to spend their free time studying rather than doing exercise [37]. The influence of these (and other possible) variables supports the importance of including a control group in which there is no intervention. When designing studies on public health issues, this type of group could be absent due to ethical criteria, operational reasons, or conceptual impossibility [42,43]. However, this control group could usefully be applied to predict the influence of biases; to enable evaluation of the results of an intervention according to the trends within the target population, reflected in the data of the control group; and to find out the factors that can influence the effectiveness of a treatment. There are strategies for reducing this impact, such as working with the trends of both groups or with the effect size of the differences between changes in averages (Cohen’s d), as carried out in one of the studies included in this review [23]. In this study, although no significant *p* value was found, it was possible to observe effect sizes, from small to medium, and this indicates that applying the intervention did have an influence. It is also important that these variables are always used with correct randomisation and an appropriate sample size to represent the target population. It may be interesting to implement future studies to investigate protocols for the use of these variables in similar studies.

Regarding the risk of bias assessment, 10 of the 13 articles included were randomised clinical trials (RCT) that obtained 3 out of 3 on the Jadad scale, except for the study by Kim et al. [22], which obtained 2 points as it did not describe the randomisation method. Questions related to blinding were not considered due to the characteristics of the interventions studied. In general, once the groups were made, there were no statistically significant baseline differences between the control group and the intervention group, so it could therefore be assumed that the differences at the end of the study were caused by the intervention. Most of the articles included analysis between the sample and withdrawal and dropout and, although those were high in some of them, they did not find significant differences between them and the remaining sample and the losses to follow-up were similar in both groups. Intention-to-treat analysis was carried out in all of them.

The present systematic review was conducted following the PRISMA checklist. Despite this, one of the limitations we find in this study is the low number of articles included in the review and the great heterogeneity of countries in which they were carried out, although the majority of the studies were RCT. Likewise, the studies were not blinded and in some cases, they show considerable losses to follow-up during the monitoring period of the study. Nonetheless, the difficulty of blinding and the losses to follow-up could be considered inherent to the characteristics of the interventions carried out. At the same time, the diverse range of countries included also represents the different ways of adapting physical activity promotion strategies in the university setting in each country. 

In view of the results of this review, educational training in physical activity promotion could be applied for students of degrees related to health as they can be implemented as part of the curriculum of the degree. This could facilitate adherence as it would be carried out within the normal class schedule. The fact that many of the studies on promotion of health and physical activity were carried out at health discipline faculties partly backs our hypothesis. In other degrees that are not health disciplines, perhaps due to the difficulty in including this material in any of the subjects except on a voluntary basis, there might not be the same acceptance, although they could be included as voluntary extra-curricular courses or implementation could imply a token increase in qualifications. On the other hand, in other strategies like incentives, the intervention will strongly depend on economic resources as the minimum amount used in studies with good results is $20 a month, which would add up to about $120 per student at the end of the semester. Future studies on cost-effectiveness are necessary to inform decisions on the implementation of strategies implemented in a university setting.

## 5. Conclusions

This systematic review describes the strategies implemented and the data collection instruments employed in physical activity promotion interventions among university students in the university environment. The eight strategies identified were used in combination and they were adapted in each of the complex interventions. Validated questionnaires were the most widely used instrument. Future original studies are needed to find out the characteristics of these measurement instruments and the impact, including health economy aspects, of these strategies on physical activity promotion among university students in the university setting. 

## Figures and Tables

**Table 1:** Descriptive characteristics of the included studies.

Author	Publication (Year)	Country	Population	Intervention Length and Measurement Times (T)
Annesi et al. [18]	2017	USA	n = 84 students. 69% women. Age (mean ± standard deviation) = 22.0 ± 5.5 years. Controlled non-randomized trial. Control group was selected from the same universities with students non-involved in the intervention.	Intervention: 10 or 15 weeks T1: Basal T2: PI ^1^ (week 10 or 15 based on the university).
Bang et al. [25]	2017	South Korea	n = 99 students and graduates from Seoul. 53% graduates 52% women. Age = 24.3 ± 4.2 years. Controlled non-randomized trial. Assignation to intervention or control group was made according to participants’ preference	Intervention: 6 weeks T1: Basal T2: PI. week 6 T3: 3 months after ending intervention.
Brown et al. [16]	2014	Canada	n = 174 first year students. 58% women Age = 17.97 ± 0.95 years Field trial. Allocation to intervention or control residence was made according to participant’s preference	Intervention: 20 weeks T1: Basal T2: Follow-up. End 2nd semester
Cameron et al. [26]	2015	United Kingdom	n = 2614 first year students 55% women. Age = 18.9 ± 2.8 years RCT ^2^	Intervention: academic year T1: Basal T2: 1 month T3: 6 months
Heeren et al. [27]	2017	South Africa	n = 176 second year students from a university in a rural area, under 25 years. 53.4% women Age = 20.84 ± 1.49 years RCT	Intervention: 4 weeks T1: Basal T2: Follow-up. 6 months T3: Follow-up. 12 months
Kattelmann et al. [19]	2014	USA	n = 1639 students from 13 universities, under 25 years. 63% women Age = 19.3 ± 1.1 years RCT	Intervention: 3 months T1: Basal T2: 3 months (PI) T3: 15 months (follow-up).
Kim et al. [22]	2018	USA	n = 187 students from a public university following a physical activity instructional program. 62% women. Age = intervention 20.32 ± 1.57 y; control 20.09 ± 1.93 y. Cluster RCT	Intervention: 15 weeks T1: Basal T2: Week 7–8 (mid-semester). T3: Week 14–15 (end of semester).
Nanney et al [23].	2014	USA	n = 1505 students from a mandatory university course about physical activity, under 25 years. 64% women Age = 19.4 ± 1.4 years, RCT	Intervention: 4 months T1: Basal T2: Week 6 (mid semester). T3: Week 12 (end of semester).
Okazaki et al. [24]	2014	Japan	n = 77 students. 35% women Age = intervention group 19.1 ± 1.3 years; control group 19.4 ± 1.2 years. RCT	Intervention: 15 weeks T1: Basal T2: 4 months (PI). T3: 12 months (follow-up).
Pope et al. [21]	2013	USA	n = 117 students from a public university. 53.8% women Age = 18 years RCT	Intervention: 12 weeks T1: Basal T2: Week 12. (end of 1st semester)
Pope et al. [20]	2015	USA	n = 117 students from a public university. 53.8% women Age = 18 years RCT	Intervention: 24 weeks T1: Basal T2: Week 12 (end of 1st semester) T3: Week 24 (end of 2nd semester)
Sharp et al. [17]	2016	Canada	n = 184 first year students. 53% women Age = 18 ± 0.69 years RCT	Intervention: 12 weeks T1: Basal T2: Week 12 (end of 1st semester)
Sriramatr et al. [28]	2014	Thailand	n = 220 female students under 25 years Age = 19 years RCT	Intervention: 3 months T1: Basal T2: Week 12. PI T3: Week 24 (follow-up)

^1^ PI: post-intervention, ^2^ RCT: randomized controlled trial.

**Table 2:** Strategies implemented and data collection tools related to physical activity employed.

Author	Intervention	Variables	Physical Activity Collection Tool
Annesi et al. [18]	INTERVENTION: Instructional elective physical activity course (25 h) including a sport-based (i.e., volleyball, tennis) or physical conditioning-based program (yoga, aerobic/strength training). CONTROL: General education course	Leisure-time PA ^1^	Godin-Shephard Leisure-Time Physical Activity Questionnaire
Bang et al. [25]	INTERVENTION: A weekly campus forest-walking program during lunchtime for 6 weeks. They were also asked, through a text message, to walk once a week additionally on an individual basis. Participants also received one lecture in small groups. CONTROL: Daily routine	-Physical activity (1) -Health promoting behaviour (2)	(1) International Physical Activity Questionnaire-Short Form (2) Health-Promoting Lifestyle Profile II (Korean version)
Brown et al. [16]	INTERVENTION: Healthy Active Living Community including structured activities based on behaviour changes techniques (interactive workshops, help, and assistance regarding organized sport teams, groups of physical exercise such as a hiking club or wall-climbing association, and challenges for healthy meals…). CONTROL: Daily routine in a community not focused on healthy active living	-MVPA ^2^ (1) -PA Action Planning (2) -PA Outcome expectancies (3)	(1) Global Physical Activity Questionnaire (GPAQ) (2) 3-items questionnaire (3) 7-items questionnaire
Cameron et al. [26]	INTERVENTION: An online theory-based intervention. Participants were asked to complete a profile page that contained the self-affirmation manipulation. Students completed four short modules on each of the four health behaviours containing theory-based messages and planning exercises. Participants had access to the full website with further health messages and educational links. CONTROL: Daily routine	Physical activity per week	International Physical Activity Questionnaire (Short-Form)
Heeren et al. [27]	INTERVENTION: 8 modules implemented during 4 weekly sessions including interactive exercises, games, role-playing, and group discussions aimed to increase physical activities, healthy diets, and limit alcohol use. Participants practiced aerobic work-out, strength building, flexibility increasing. CONTROL: Same number of sessions focused on HIV ^3^ risk reduction	Physical activity during the last week	3 open-ended items to establish if the participant met the physical activity guidelines
Kattelmann et al. [19]	INTERVENTION: 21 mini-educational lessons and e-mail messages about eating behavior, physical activity, stress management, and healthy weight management. Implemented through a personalized website and following precede-proceed model. Participants visit the website weekly to set goals, view a graph of their goal and recommendations. During the follow-up phase, website and e-mail remained active but no new lessons were added. CONTROL: Daily routine	Physical activity per week	International Physical Activity Questionnaire
Kim et al. [22]	INTERVENTION: Activity tracker was provided to be used daily during the semester and it was linked to an app for smartphones. It provided physical tracking, goal setting, and behavioural feedback, among others. CONTROL: Daily routine	Physical activity per week	Uniaxial accelerometer during 7 days in each measurement time
Nanney et al. [23]	INTERVENTION: Instructors of PA course received a need-supportive training during 60-min weekly. Two subgroups were built: one subgroup used a pedometer daily (linked to an app to check their goals and set new goals). The other subgroup used the pedometer just to collect information in three specific weeks (basal mid-term and end of semester). CONTROL: Instructors received conventional training during 60-min sessions weekly	-PA (1, 2, 3) -Steps/day (4) -PA enjoyment (5) -State of Change (6) -Motivation to be active (7)	(1) International Physical Activity Questionnaire-Short Form (IPAQ-SF) (2) 30-Day Physical Activity Recall (3) 8-response Physical activity self-report measure (4) Pedometer (5) 5-item Exercise enjoyment scale (6) 4-item about Physical Activity State of Change (7) Behavioral Regulation in Exercise Questionnaire-modified
Okazaki et al. [24]	INTERVENTION: Internet-based PA education course also with four face-to-face sessions. Participants set their goals and a weekly schedule that could be modified by them. Once a week, they received a message and a web-based quiz about physical activity, exercise, and other healthy lifestyles. CONTROL: Non-health related course during the study	-Physical activity per week (1) -State of Change (2)	(1) International Physical Activity Questionnaire (2) Stages of Change Scale for physical activity
Pope et al. [21]	INTERVENTION: Weekly monetary incentives during the first semester based on escalating rewards and reset contingency. During week one, they received $5 dollars for each 30-min gym visit. Every week, this amount increased by $0.25 per visit (up to max. of $7.75) as the required number of visits also increased. If they failed to reach the goal, the amount returned to base. They had access to a website displaying average and potential amount. CONTROL: No monetary payments for same goals	Gym center attendance	Identification electronic card
Pope et al. [20]	INTERVENTION 1: Continued-incentive condition receiving weekly incentives during the fall semester and incentives on a variable-interval schedule during the spring semester ($40 in four random weeks unknown to the participants). The fitness-center attendance was five 30-min visits per week. INTERVENTION 2: A discontinued-incentive condition receiving weekly incentives during the fall semester and no incentives during the spring semester CONTROL: No monetary payments for same goals	-Gym center attendance (1) -Motivation to be active (2)	(1) Identification electronic card (2) Exercise motivation inventory-2 (51-items) (EMI-2)
Sharp et al. [17]	INTERVENTION: Pedometer-based intervention. Participants were asked to wear the pedometer daily during the study and record a step log calendar. They received three monthly e-mails reminding them to record their steps and which provided tips and opportunities to increase their physical activity on campus and some health promotion educational information. CONTROL: Usual daily routines	-Physical activity (1) -Leisure time PA (2)	(1) Pedometer (2) Modified Godin-Shephard Leisure-Time Physical Activity Questionnaire.
Sriramatr et al. [28]	INTERVENTION 1: SOC theory-internet intervention with pre-test. Participants received a pedometer and accessed the website to record their physical activity, set goals for the next week, and identify expectative and self-efficacy. Weekly e-mails were sent, reminding them to visit the website and giving personal feedback and providing physical activity information. Participants were encouraged to accumulate at least 90 min of MVPA per week and to increase by 9 min/week. INTERVENTION 2: Intervention without pre-test: same intervention excluding pre-test CONTROL 1: Pre-test CONTROL 2: Daily usual routine (no pre-test)	-Leisure time PA (1) -Steps/day (2) -SOC ^4^ variables (3)	(1) Godin-Shephard Leisure-Time Physical Activity Questionnaire (Thai version) (2) Pedometer (3) Outcome Expectations; Multi-dimensional Self-Efficacy for Exercise Scale; Self-Regulation Questionnaire

^1^ PA: Physical activity ^2^ MVPA: Moderate-to-vigorous Physical activity ^3^ HIV: Human immunodeficiency virus ^4^ SOC: Social Cognitive Theory.

## Supplementary Materials

The following are available online at https://www.mdpi.com/1660-4601/17/18/6526/s1, Table S1: Strategies used to promote physical activity in included studies. Table S2: Impact of the interventions implemented in included studies.
