# Supplementary material for: Strategies and Measurement Tools in Physical Activity Promotion Interventions in the University Setting: A Systematic Review
Source: Int J Environ Res Public Health. 2020 Sep 8;17(18):6526. doi: 10.3390/ijerph17186526 (PMC7557565; doi:10.3390/ijerph17186526)
Supplement: Supplementary file 1 [file ijerph-17-06526-s001.pdf]

Table S1. Strategies used to promote physical activity in the included studies.

	Health Promotion Course (online or face-to face)	Sending Periodic Messages	Online Profile Creation (web or APP)	PA[1], Exercise, or Sports Programs Attendance	Pedometer or Activity Tracker	Incentives to Attend Fitness Center	Training of PA Courses Teachers	Pre-Test Sensitization
Annesi et al. [16]	■			■				
Bang et al. [23]	■	■		■				
Brown et al. [14]	■			■				
Cameron et al. [24]	■	■	■					
Heeren et al. [25]	■			■				
Kattelmann et al. [17]	■	■	■					
Kim et al. [20]			■		■			
Nanney et al [21]			■		■		■	
Okazaki et al. [22]	■	■	■					
Pope et al. [19]						■		
Pope et al. [18]						■		
Sharp et al. [15]	■	■			■			
Sriramatr et al. [26]	■	■	■		■			■

[1] Physical activity

Table S2. Impact of the interventions implemented in included studies.

	Variables	Between Groups Differences *	Within Intervention Group Differences *	Within Control Group Differences *
Annesi et al. [18]	Leisure-time PA[1]		Yes	Yes
Bang et al. [25]	- Physical activity<br>- Health promoting behaviour	No (PA)<br>Yes (health promoting behaviour)		
Brown et al. [16]	-MVPA[2]<br>-PA Action Planning<br>-PA Outcome expectancies	Yes (MVPA and PA action planning)		
Cameron et al. [26]	Physical activity per week	No		
Heeren et al. [27]	Physical activity during the last week.	Yes (vigorous PA at 6 months)<br>Yes (moderate PA and vigorous PA at 12 months)		
Kattelmann et al. [19]	Physical activity per week.	Yes (vigorous PA in women for 3-months)	Yes (moderate PA at 3 months and 15 months)	Yes (moderate PA at 3 months and 15 months)
Kim et al. [21]	Physical activity per week	No	Sedentary times increment<br>Light intensity PA reduction.	MVPA reduction
Nanney et al [23].	- PA<br>- Steps/day<br>- PA enjoyment<br>- State of Change<br>- Motivation to be active	No (main study)<br>Yes (steps/days on the substudy)	Increment of PA from time 0 to mid-semester.<br>Reduction of PA from mid-semester to end of semester.	
Okazaki et al. [22]	- Physical activity per week<br>- State of Change	Yes (energy expenditure on non-engaged in sports subgroup in the intervention group)<br>Yes (State of change)	Yes (state of change)	Yes (state of changes on subgroup engaged in sports)

Pope et al. [21]	Gym center attendance	Yes	Reduction across the semester	Reduction across the semester
Pope et al. [20]	- Gym center attendance<br>- Motivation to be active	Yes (during the fall semester, between the control condition and the incentive conditions)<br>Yes (during the spring semester, between the continued-incentive group and the other two groups)		
Sharp et al. [17]	-Physical activity<br>-Leisure time PA	No	Mild PA increment<br>Vigorous PA reduction	Mild PA increment<br>Vigorous PA reduction
Sriramatr et al. [28]	- Leisure time PA<br>- steps/day<br>-SOC[3] variables	Yes (steps/ day, leisure time physical activity and SOC variables at the 3 and 6 month follow-up)<br>No (for pretest sensitization)		

*statistically significant [1] PA: Physical activity [2] MVPA: Moderate-to-vigorous Physical activity [3] SOC: Social Cognitive Theory
